# The University Münster model surgery system for Orthognathic surgery. Part I – The idea behind

**DOI:** 10.1186/1746-160X-8-14

**Published:** 2012-05-14

**Authors:** Ulrike Ehmer, Ulrich Joos, Stefanie Flieger, Dirk Wiechmann

**Affiliations:** 1 Department of Orthodontics, University of Münster, Albert-Schweitzer-Campus 1, 48149, Münster, Germany; 2 Department of Maxillofacial Surgery, University of Münster, Albert-Schweitzer-Campus 1, 48149, Münster, Germany; 3 Department of Orthodontics, Medizinische Hochschule Hannover, Carl-Neuberg-Str. 1, 30625, Hannover, Germany

## Abstract

**Background:**

We describe a procedure for diagnosis and planning for orthognatic surgery based on international standards. A special 2D planning based on lateral cephalograms (Axis Orbital Marker Lines System) realize a transmission to the SAM 2P articulator (3D) by means of the Axis Orbital Plane.

**Methods:**

Former intraoperative measurement of the average height of the LeFort I osteotomy plane relative to the molar occlusal plane allow to construct a virtual osteotomy plane in the lateral cephalogram. This is the basis for the development of the Axis Orbital Marker Lines System (AO-MLS).

**Results:**

The AO-MLS is presented graphically, and in detail, with construction guidelines. The system could be integrated into various lateral cephalometric analysis- and surgical prediction schemes. It forms the basis for a standardized transfer of the 2D planning to the 3D planning in the articulator, and vice versa. This procedure makes it possible to generate surgical planning protocols based on the model surgery, which represent the dislocations in the proximity of the real osteotomy planes.

**Conclusions:**

The Axis Orbital Marker Lines System (software component) in conjunction with the University Münster Model Surgery System (hardware system) increases the predictability of model operations in orthognathic surgery.

## Background

Our approach for diagnosis and treatment planning complies to international standards for Combined Surgical Orthodontic Treatment [1-8].

**Figure 1:**
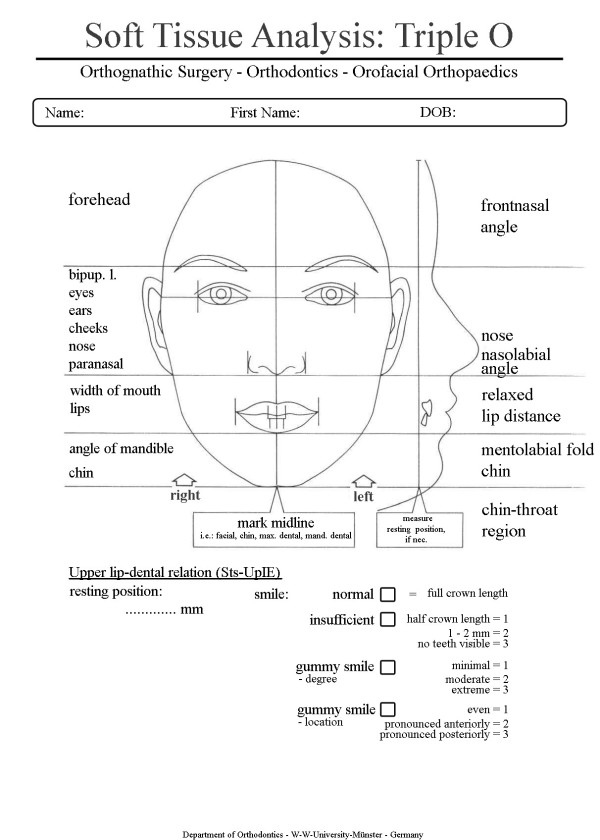
Graphically supported clinical examination form.

The diagnostic procedure comprises modified standard procedures of collecting the patient’s medical history, comprehensive clinical examinations, facial analysis using a custom-designed form for a concise clinical documentation (Figure 1), taking impression for plaster models, x-rays, and customized analysis- and documentation procedures, developed from international analysis- and documentation procedures combined with our own measurement modifications.

The psycho-social status of the patient is being assessed preliminarily by means of a psychological screening interview (PSI) [9-12], following a “traffic-light”- approach: a positive status comprises mainly of answers which are highlighted in green, whereas the indication for a more detailed psychological evaluation is given by answers mainly from the red highlighted spectrum.

**Figure 2:**
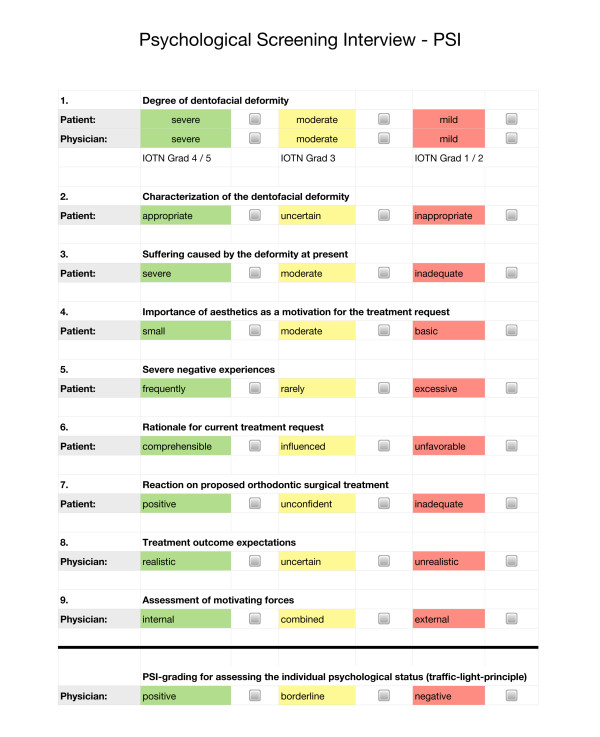
**Preliminary assessment of the psychosocial status for orthognathic surgery by means of a psychological screening interview (PSI) using the “traffic light principle”.** The PSI is being made during the patient interview and amended by the IOTN items: degree of the dentofacial deformity. An expected positive status consists of a predominantly green marked response spectrum, whereas a mostly red marked response spectrum is a warning signal and is seen as an indication for further psychological examination.

Figure 2 shows this color-coded screening protocol. In addition as a part to the basic PSI, the severity of the dento-facial deformity (IOTN) is being reviewed [13].

The medical indications and contra-indications and the combined pre- and post-surgical orthodontic sequences, as well as informing the patient about the necessary treatment and the risks, will be discussed in an interdisciplinary initial appointment, and also during a follow-up appointment, where modifications can be made according to the case.

Once the pre-surgical orthodontic treatment has been deemed to be satisfactory, the next step is to obtain the necessary records for the final surgical planning: dental casts, mounted in a semi-adjustable articulator (SAM 2P, Schul-Artikulator-München, SAM Präzisionstechnik, Gauting, Germany), using a face-bow-transfer, panoramic x-rays, lateral cephalograms, fotos, and if indicated posterior-anterior cephalograms, cone beam computer tomograms, hand wrist x-rays.

**Figure 3:**
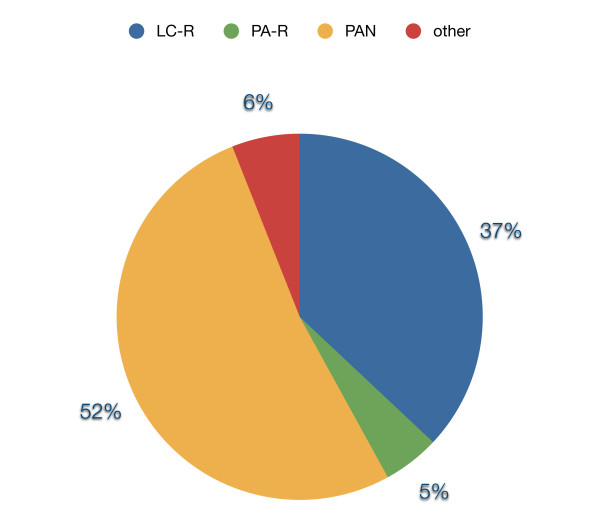
**Distribution of the different types of x-rays (1993–2001).** LC-R (lateral cephalometric radiographs), PA-R (posterior-anterior radiographs), PAN (panoramic radiograph).

Figure 3 gives an overview about the frequencies of occurrence of the different types of x-rays that have been taken of the patients who attended the Department of Orthodontics at the University of Münster [14].

**Figure 4:**
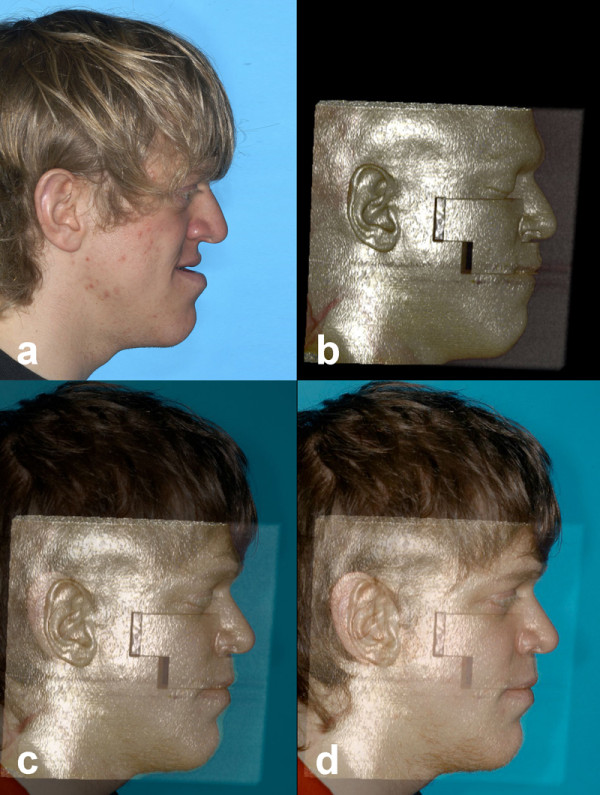
**Example of computer-aided surgery (CAS) of a patient with Crouzon syndrome.** Simulation and result of a Le Fort II distraction. **a**) before surgery, **b**) CT planning, **c**–**d**) different superimpositions of the CT planning with the final result.

The information for the necessary surgical displacement is generated in the cephalometric prediction schemes, using a special analysis protocol for lateral cephalometric radiographs. The two-dimensional cephalometric prediction planning in the sagittal and vertical dimension is being completed by transversal data from the model analysis, and by the posterior-anterior analysis in cases with asymmetry. Only in 1,4% of our cases of orthognathic surgery, we have integrated 3D-methods based on computer tomography, stereolithographic models or computer aided surgery (Figure 4).

**Figure 5:**
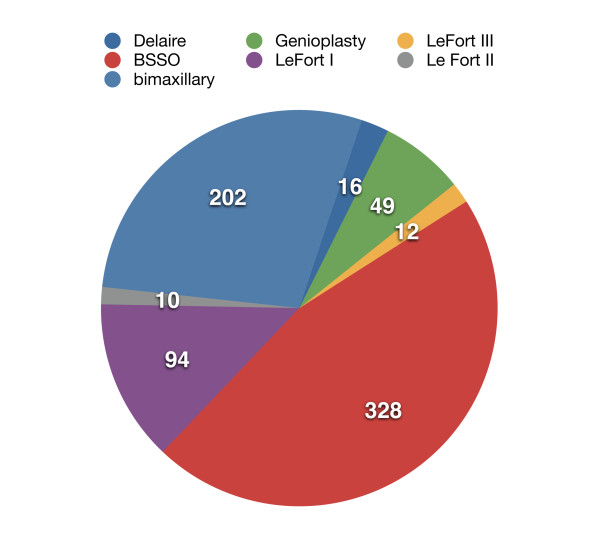
Surgical procedures (absolute numbers) for the period 2004–2008, compiled to the German Operation Coding System (OPS).

The differentiated spectrum of surgical procedures in our Department of Maxillofacial Surgery is shown graphically in Figure 5.

Compared to international literature, there are some differences in the spectrum of surgical techniques, due to specific techniques that are preferred in Muenster as well as ethnic differences in malocclusion prevalence.

## Methods

**Figure 6:**
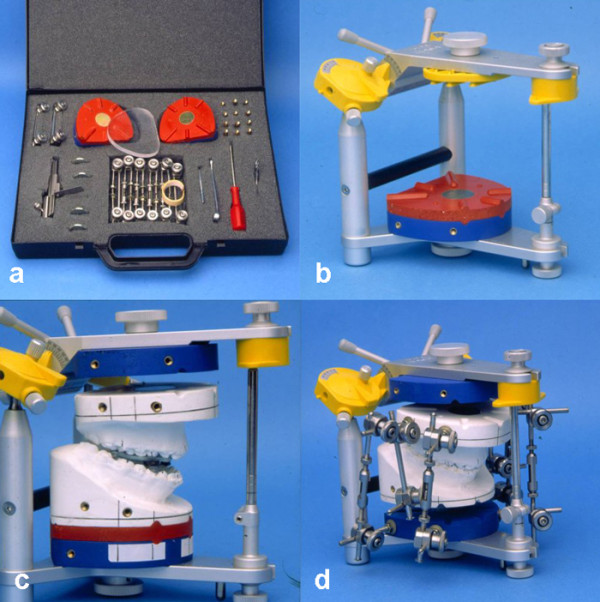
**Overview of the KD-MMS. a**) hardware of the system, **b**) double split plates added to the lower part of the SAM-P, **c**) models mounted and ready for surgical maxillary displacement. The whole system is oriented parallel to the Axis Orbital plane. **d**) final position before fixation with removable plaster casts.

For the final surgical planning, Ehmer et al. [15,16] have developed a differentiated model surgery system: Calibrated Double splint - Münster Model Surgery System (German abbreviation: KD-MMS). An overview is presented in Figure 6. The development process was not based on experimental research carried out on humans or animals. Written informed consent was obtained from the patient for publication of this report and any accompanying images.

In the process, an Axis Orbital Marker Lines System (AO-MLS), which could be integrated in every planning process, and a SAM 2P articulator compatible model surgery system, have been combined.

From former intraoperative measurements of the average height of the LeFort I osteotomy plane relative to the molar occlusal plane, allows to construct a virtual osteotomy plane in the lateral cephalogram (ML-2). This is the basis for the development of the Axis Orbital Marker Line System (AO-MLS).

## Results and discussion

The Axis Orbital Plane is defined as being the common reference plane for the lateral cephs and the articulator-based model surgery in a couple of schemes [17-19]. However, in the Münster model scheme, it has been further developed into a structured combination between 2D and 3D predictions in the model surgery.

**Figure 7:**
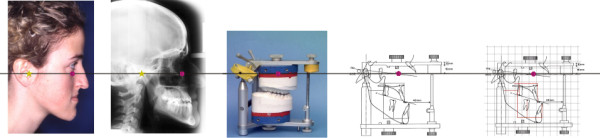
The Axis Orbital Plane is the basis for measurement transfer between two-dimensional (lateral ceph) and three-dimensional (articulator with mounted models) planning records.

Figure 7 shows the common reference plane (Axis Orbital Plane) of the whole concept. This reference plane is constructed in the lateral cephalometric tracing by rotationg the Frankfort Plane by 7 degrees caudally around the orbital landmark. For complex cases it’s possible to transfer an individual hinge axis into the lateral ceph using metal-markers. This common reference is being used by a couple of other systems [17-19]. However, in the present system it is being developed into a structured link between the two-dimensional and three-dimensional predictions.

Based on this reference plane, the custom software was developed using the Axis Orbital Marker Lines System (AO-MLS). The AO-MLS could be be integrated into every cephalometric analysis-prediction system. When transferring the data, it is imperative to maintain a 1:1 ratio, which is a precondition in every prediction system.

**Figure 8:**
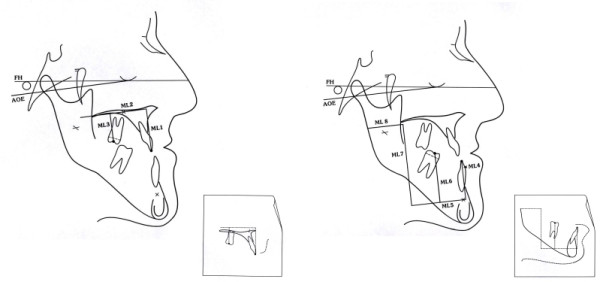
**Axis Orbital Marker Lines System (AO-MLS). The reference plane is based on the Axis Orbital Plane (AOE).** Construction: Rotation of the Frankfort Plane caudally by 7° around the orbital landmark. Left: Marker lines of the maxilla. -ML1 from the incisal landmark of the upper central incisor perpendicular to ML2. -ML 2 approx. 5 mm above the mesial apex landmark of the upper first molar, parallel to the Axis Orbital Plane (first construction step). -ML3 from the buccal fissure (alternatively buccal mesial cusp) of the first upper molar perpendicular to ML2. Right: Marker lines of the mandible. -ML4 from the lower central incisor edge perpendicular to ML5. -ML 5 approx. 5 mm below the apex of the lower central incisor, parallel to the Axis Orbital Plane (first construction step). -ML 6 from the buccal fissure (alternatively buccal mesial cusp) of the first lower molar perpendicular to ML5. -ML 7 from the anterior margin of the ramus perpendicular to ML 5. -ML 8 above the Lingula mandibulae, parallel to the axis-orbital plane. The small boxes show the duplication of the planes and of the maxillary and mandibulary structures as a template for the displacement according to vertical and sagittal targets for the surgical planning.

The three maxillary lines (Figure 8 left, ML1-3) follow the virtual line of osteotomy either parallely or perpendicularly and are oriented parallel or perpendicular to the Axis Orbital Plane. The five mandibular lines approximately represent the area of the sagittal splitting of the lower jaw and are complemented by dental references (Figure 8 right, ML4-8).

The eight planes are being transferred to the cephalometric tracing and to an overlayed second tracing (template) either by hand or by a computer analysis software. The templates for the upper and lower jaws (bimaxillary surgery) will be moved to the sagittal and vertical targets. In monognathic surgery this will be done isolated for the upper or lower part.

The displacements in the region of the osteotomy can now be measured with some accuracy by means of the marker lines. The difference between the MLs on the ceplalometric tracing to the overlayed template represent the skeletal effects. The difference of the dental landmarks represent the dental displacements. Comparing the dental and skeletal movements could be helpful in determining rotational effects or for borderline movements.

Additionally, it is possible for example to visually point out different rotations in the dental region and in the region of osteotomy. During the procedure of planning, we can identify desired or unfavorable rotations and subsequently check their feasibility in the three-dimensional planning.

**Figure 9:**
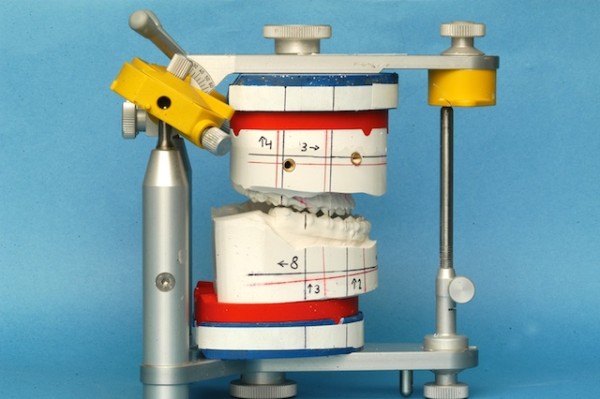
**With a modified parallelometer perpendicular and horizontal lines (ML1-ML8) could be traced on the mounted plaster models.** The horizontal marker lines are drawn in their individual heights obtained from the cephalometric analysis. The vertical marker lines are oriented according to the dental landmarks on the plaster model. Alternatively to the parallelometer this orientation can be found with a simple ruler (triangle), with little compromise in accuracy and more time consuming. After model surgery the lines in the new position (postoperative) have to be drawn, to get the 3D skeletal displacement.

In the articulator mounted and perpendicular trimmed models only dental parameters are being defined. With modified parallelometer which is parallel or perpendicular orientated to the articulator reference plane (Axis Orbital Plane) the marker lines could be constructed on the mounted plaster models (skeletal reference). The construction of the Axis Orbital Marker Lines System (AO-MLS) should be done according to their individual heights and distances, measured from the lateral ceph. Figure 9 demonstrates the procedure in a clinical case.

The individual user may modify this system of lines, maintaining the basic principle.

## Conclusions

The AO-MLS (software component) in combination with the KD-MMS (hardware system) increases the predictability of model operations in orthognathic surgery.

## Competing interests

The authors declare that they have no competing interests.

## Authors’ contributions

UE developed the KDMMS and suggested the original idea for the paper. UE and SF wrote the main part of the manuscript. UJ and DW reviewed the paper for content, and reviewed and contributed to the writing of all iterations of the paper, including the final version of the manuscript. All authors read and approved the final manuscript.
